# Pyroptosis-Related lncRNAs for Predicting the Prognosis and Identifying Immune Microenvironment Infiltration in Breast Cancer Lung Metastasis

**DOI:** 10.3389/fcell.2022.821727

**Published:** 2022-03-04

**Authors:** Li Liu, Chenxi Chen, Gang Tu, Yang Peng, Meiying Shen, Yingkun Xu, Shengchun Liu

**Affiliations:** ^1^ Department of Endocrine and Breast Surgery, The First Affiliated Hospital of Chongqing Medical University, Yu-Zhong, China; ^2^ Department of Geriatrics, The First Affiliated Hospital of Chongqing Medical University, Yu-Zhong, China

**Keywords:** pyroptosis, breast cancer, immunotherapy, prognosis, lncRNA

## Abstract

Breast cancer (BC) is the second leading cause of death among women and is highly heterogeneous. Three pyroptosis (PR) subtypes were identified in patients with BC from the Cancer Genome Atlas Database (TCGA) cohorts using 20 PR-related regulators, which illustrate a strong association between BC prognosis and PR. Lung metastasis commonly occurs in the advanced stages of BC, resulting in a poor quality of life. Eight differentially expressed (DE) lncRNAs were identified using LASSO–Cox analysis between PR-related and BC lung metastasis. Moreover, a BRCA risk-score (RS) model was established using multivariate Cox regression, which correlated with prognosis in TCGA-BRCA. Clinical characteristics, tumor mutation burden, and tumor immune cell infiltration were extensively investigated between high- and low-RS groups. Similarly, a lower RS implied longer overall survival, greater inflammatory cell infiltration, and better immunotherapeutic response to *PD-1* blockers. Our findings provide a foundation for future studies targeting PR and confirme that RS could predict the prognosis of patients with BC.

## Introduction

Breast cancer (BC) remains a major public health problem worldwide and is the second leading cause of cancer death among women after lung cancer. The 5-years overall survival (OS) rate of patients with BC diagnosed from 2009 to 2015 was 98, 92, 75, and 27% for stages I, II, III, and IV, respectively (Bray et al., 2018; Siegel et al., 2021). According to the International Agency for Research on Cancer, >2.26 million new cases of BC and approximately 685,000 deaths from BC were reported globally in 2020 (https://publications.iarc.fr/Non-Series-Publications/World-Cancer-Reports/World-Cancer-Report-Cancer-Research-For-Cancer-Prevention-2020). BC accounted for >10% of all new cancer cases and approximately 7% of all cancer deaths in 2020 (Siegel et al., 2021). Metastasis remains the primary threat to the lives of patients with cancer with only a few effective therapeutic options, and metastasis of breast cancer (MBC) is the leading cause of cancer-related morbidity and mortality among women worldwide. The prognosis for patients with MBC is poor, with the 5-years survival rate of only 26% (Lu et al., 2009). Furthermore, lung metastasis is the major site associated with the mortality of patients with MBC (Solomayer et al., 2000). Therefore, it is imperative to establish effective prognostic models for predicting the OS of patients with MBC as guidelines in clinical practice.

Pyroptosis (PR), a new form of programmed cell death and also known as cell inflammatory necrosis (Shi et al., 2016), was firstly characterized in myeloid cells infected by pathogens or bacteria in 1992 (Zychlinsky et al., 1992; Daly et al., 2020). The PR-related cells are characterized by cell swelling and numerous bubble-like vesicles under the electron microscope. Small pores are formed on the cell membrane that releases inflammatory cytokines (Bauer et al., 2007; Miao et al., 2010; Wang et al., 2017; Chen et al., 2020). PR not only antagonized infection but also correlated with cancer progression. Inflammatory vesicles, gasdermin proteins, and pro-inflammatory cytokines have been reported as key components of PR and were associated with tumor initiation, invasion and metastasis (Miao et al., 2010; Liu et al., 2016; Zhou et al., 2020). Dupaul-Chicoine *et al.* knocked out inflammatory vesicle-associated genes (NLRP3 and CASP1) in transgenic mice and found that these mice were more likely to develop colon cancer compared with mice with a wild-type version (Dupaul-Chicoine et al., 2010). Moreover, various danger-related signaling molecules and cytokines, inflammatory response, and immune system (Tang et al., 2020) are activated and released when PR occurs (Miao et al., 2010).

Previous studies have confirmed that the effects of pro-inflammatory PR are caused by the regulation of the tumor immune microenvironment (Erkes et al., 2020), and we found that GSDMD defective expression was associated with a significantly decreased number and activity of CD8 + T lymphocytes (Xi et al., 2019). A recent study has also confirmed the pivotal role of PR in the anti-tumor function of NK cells (Zhang et al., 2020b). Ping, Liqin and Lv W et al. also demonstrated that PR-associated lncRNAs serve a prognostic role in breast cancer as well as its correlation with TME (Lv et al., 2021; Ping et al., 2021). Therefore, PR plays an important role in tumor development and the anti-tumor process (Wu et al., 2021); however, its specific function in BC remains to be further elucidated. Therefore, the immune cell infiltration characteristics of the TME with identified PR regulators should be comprehensively estimated to enhance our understanding of tumor immunity and anti-tumor inflammatory response.

In the present study, we aimed to establish a scoring model (that produced the risk score [RS]) by classifying patients with BC based on PR-related lncRNAs to predict the prognosis and guide the clinical treatment. Furthermore, the RS model was used to assess the clinical characteristics, tumor mutation burden (TMB) and tumor immune cell infiltration among different RS groups of BRCA. Our findings reveal the potential association among PR, prognosis, immune microenvironment and response to immunotherapy of patients with MBC.

## Materials and Methods

### Dataset Source and Pre-Processing

Public gene expression data and complete clinical annotations were searched in the Gene Expression Omnibus Database (GEO) and the Cancer Genome Atlas Database (TCGA). In TCGA datasets, RNA sequencing data of gene expression (FPKM values) and clinical information were obtained from UCSC Xena (https:Fig.//gdc.xenahubs.net). The GSE96058 gene expression matrix file was downloaded from the GEO database (http://www.ncbi.nlm.nih.gov/geo/), and Illumina probe sequences were obtained from annotation files GPL18573 and GPL11154. GSE96058 data and TCGA-BRCA were processed in the following steps: 1) samples without clinical follow-up information were removed, 2) samples with unknown survival time, <0 days and no survival status were removed, 3) probes were converted to gene symbol, 4) the probe corresponding to multiple genes was removed and 5) the median value with multiple expressions of a gene symbol was noted. After processing, a total of 113 paracancerous and 1,082 cancer tissue samples were included from TCGA-BRCA, and 3,409 cancer samples were included from GSE96058. A total of 20 pyroptosis-related genes (PRGs) were identified using a “pyroptosis” keyword search in the Gene Cards database (https://www.genecards.org/) and validated in several articles (Supplementary Material/Supplementary Tables S1, S2) (Schroder and Tschopp, 2010; Shi et al., 2016; Fang et al., 2020; Hartman, 2020).

### Unsupervised Clustering of PRGs

To elucidate the biological properties of PRGs in BRCA, the “Consensus ClusterPlus” package (Wong, 1979) (1,000 iterations and 80% resampling rate, http://www.bioconductor.org/) was used to divide patients with BRCA into different subtypes for further analysis. The stability and patterns of molecular clusters were adjusted by the consensus clustering algorithm (Wong, 1979). The “Consensus ClusterPlus” package was employed to cluster, and the process was performed 1,000 times (Wilkerson and Hayes, 2010). Thereafter, gene expression patterns were assessed using PCA analysis. Kaplan–Meier analysis was performed to compare the OS between the high- and low-risk groups in the validation set.

### Differential Expression Analysis Among Subtypes

Tumor samples were classified into different subtypes after a consistent clustering of PRG expression. Differentially expressed genes (DEGs) among subtypes were obtained using the “limma” package (Ritchie et al., 2015). The significance criteria for selecting lncRNAs was set at adjusted *p <* 0.05 and an absolute value of log2 FC ≥ 1 and annotated with genome file (*. GTF) using Ensemble.

### Construction of a PR-Related lncRNA RS Model

We constructed an RS model for tumors based on PR-related lncRNAs. Univariate cox analysis was performed on these PR-related lncRNAs to identify lncRNAs associated with the survival of patients. Subsequently, the least absolute shrinkage and selection operator (LASSO) algorithm was used to reduce dimensionality. Finally, multivariate cox analysis was performed based on lncRNAs after dimensionality reduction to create a prognostic prediction model of tumor immune cell infiltration. The calculation formula was as follows:

Risk_scores=∑Coef(i)∗Exp(i)

### Estimation of TIME Cell Infiltration

We used the single-sample gene set enrichment analysis (ssGSEA) algorithm to quantify the relative proportions of immune cells. Each TIME-infiltrating immune cell type was labeled as described in a study by Charoentong, including multiple subtypes of human immune cells such as activated CD8 T cells, dendritic cells, macrophages, NK T cells and regulatory T cells [14, 15]. The ssGSEA algorithm was used to quantify the immune microenvironment by calculating the abundance of each TIME-infiltrating cell.

### Gene Set Variation Analysis

GSVA analysis was used to explore the biological pathway difference between high- and low-risk patients. “c2.cp.kegg.v7.2.” and “symbols” were downloaded from the MSigDB database for GSVA analysis. The R package “limma” was used to calculate differentially expressed pathways (Ritchie et al., 2015). FDR < 0.05 and | log2FC | > 0.1 were considered significant.

### Construction and Verification of Prediction Graph

A prognostic nomogram was created to predict 1-, 3-, and 5-years OS for patients with BRCA in the TCGA dataset using all prognostic and clinical parameters, which are tested for collinearity using the Cox regression model. The nomogram calibration curve was drawn to compare the predicted and observed OS.

### Collection of Immune Checkpoint Blockade Genomic and Clinical Information

A systematic search of blocking gene expression profiles of immune checkpoints, which are publicly available for clinical information, was performed. We eventually included an immunotherapy cohort, a cohort of advanced renal clear cell carcinoma treated with PD-1 blockade (also known as the PD-1 blocking cohort). For this cohort, complete expression data and detailed clinical annotations are available as supplemental material (https:// www.ncbi.nlm.nih.gov/pmc/articles/PMC7499153/). Finally, 251 samples were selected for further analyses after excluding samples with incomplete treatment response information.

### Statistical Analysis

All statistical analyses were performed using R version 3.5. Differences in expression levels of PR-related factors between TP53 and PIK3CA mutant and wild-type samples were compared using the Wilcox test. Survival curves were generated using the Kaplan–Meier method, and differences were compared using the log-rank test. Univariate and multivariate analyses were performed using Cox regression models to determine the prognostic RS and clinical features. ROC curves were used to estimate the predictive efficiency of the risk model for 1-, 3-, and 5-years OS. *p* < 0.05 indicated statistical significance.

## Results

### Molecular Characterization of Genes Involved in BC PR

**FIGURE 1:**
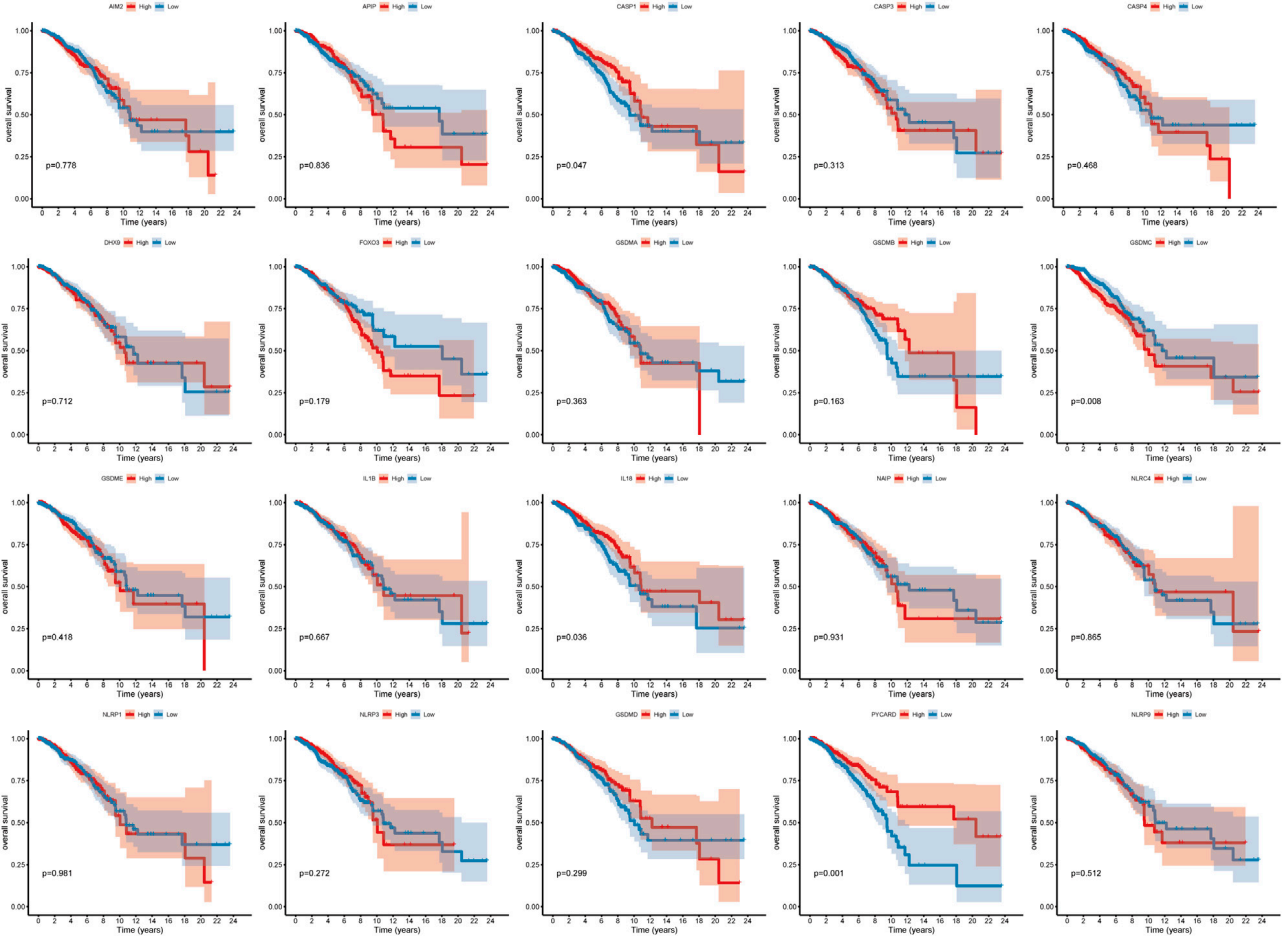
Survival curve of pyroptosis-related genes.

**FIGURE 2:**
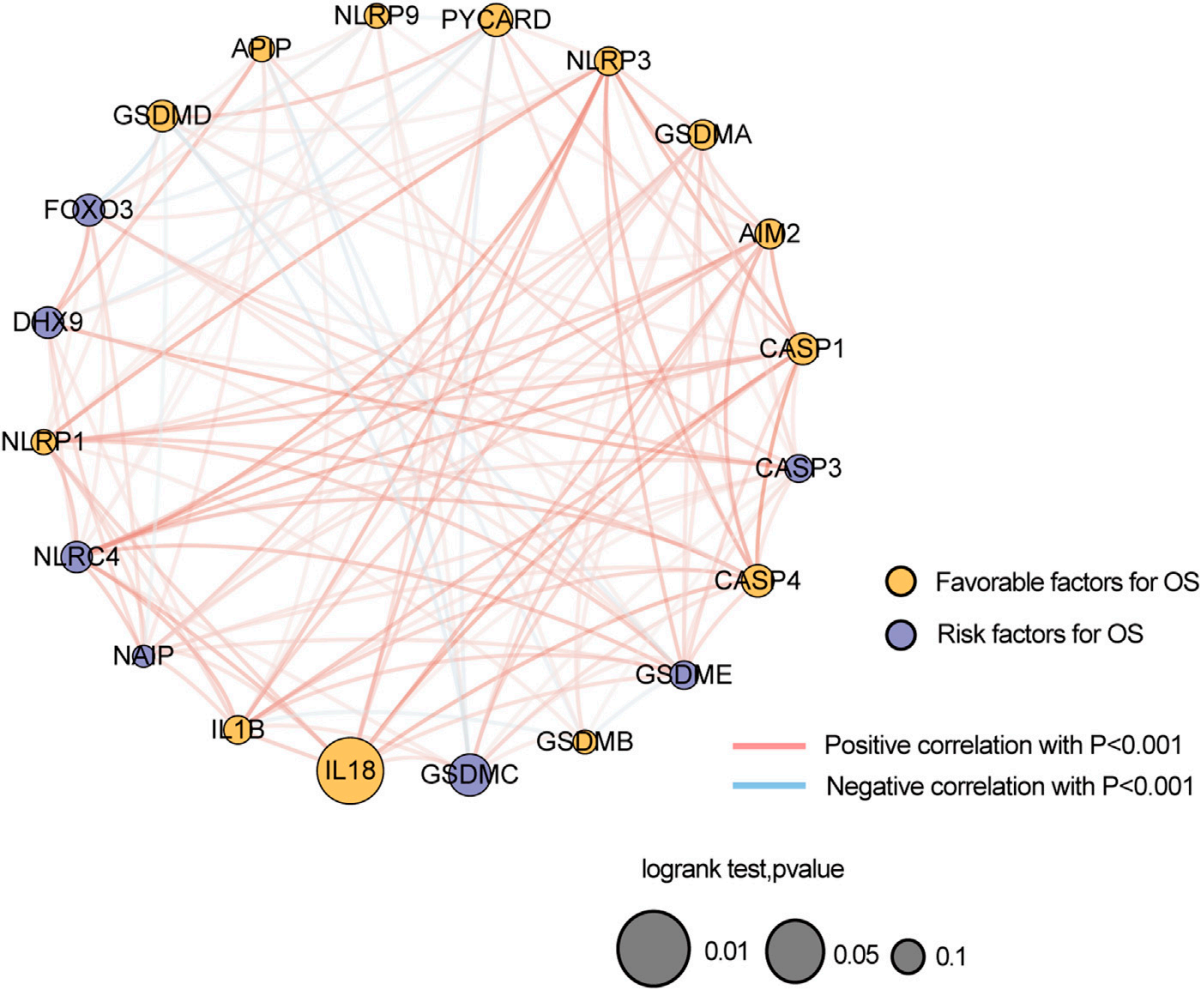
Correlation network of the expression of 20 pyroptosis-related genes in TCGA-BRCA dataset.

A total of 20 PR genes were included in this study from TCGA cohorts (Supplementary Tables S1, S2). According to median expression, samples were separated into two groups: high- and low-expression. High expression of CASP1, IL18, and PYCAPD and low expression of GSDMC were significantly associated with a better prognosis (Figure 1). Subsequently, gene mutations in the TCGA-BRCA dataset were counted, and 88.74% of the tumor samples had gene mutations, with TP53, PIK3CA, TTN, CDH1, and GATA3 having a relatively high proportion of mutations, i.e. 34, 33, 16, 13, and 12%, respectively (Supplementary Figure S1). Of these, TP53 and PI3K were the leading mutations. To ascertain whether the expression level of PR genes is associated with TP53 and PIK3CA mutation groups, the expression of 20 PR genes was investigated in both mutation samples. The results (Supplementary Figure S2) indicated a positive correlation of TP53 mutation with high expression of AIM2, CASP1, CASP3, GSDMB, GSDMC, GSDME, IL1B, IL18, and NLRC4 and low expression of GSDMD, NAIP, NLRP1, NLRP3, and PYCARD. Additionally, PIK3CA mutation was positively correlated with high expression of CASP1, CASP4, FOXO3, GSDME, IL18, NAIP, NLRP3, and PYCARD while correlated with low expression of GSDMB (Supplementary Figure S3). In addition, we analyzed the correlation network of OS in TCGA-BRCA datasets with 20 PR regulators (Figure 2).

### Identification of Pyroptotic Subtypes and Differentially Expressed Genes in BC

**FIGURE 3:**
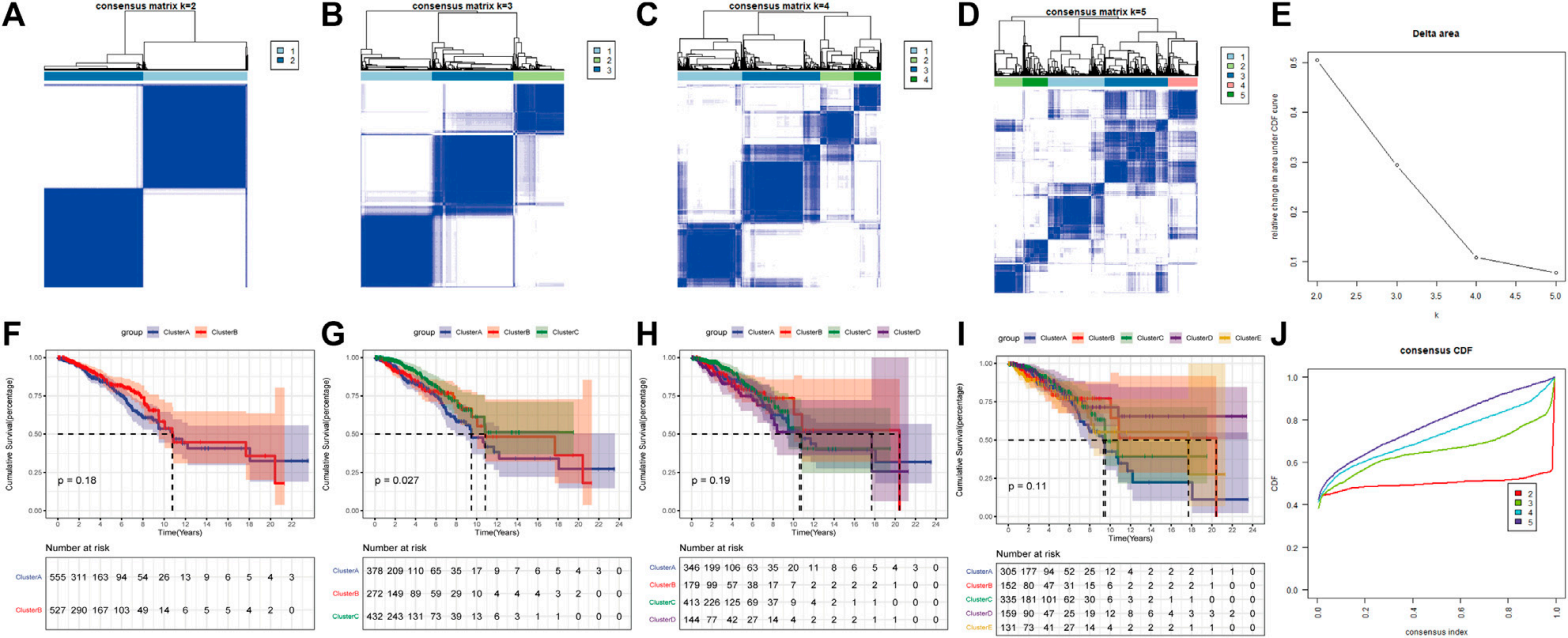
Consensus clustering of pyroptosis-related regulators in Breast cancer by the k-means method. **(A–D)** Consensus clustering of 20 pyroptosis phenotype-related genes in TGGA-BRCA cohorts and consensus matrices for k = 2–5. **(E)** The relative change area under CDF curves for K = 2–5. **(F–I)** Survival curve for K = 2–5. **(J)** The consensus CDF curves were shown for different k from 2 to 10.

**FIGURE 4:**
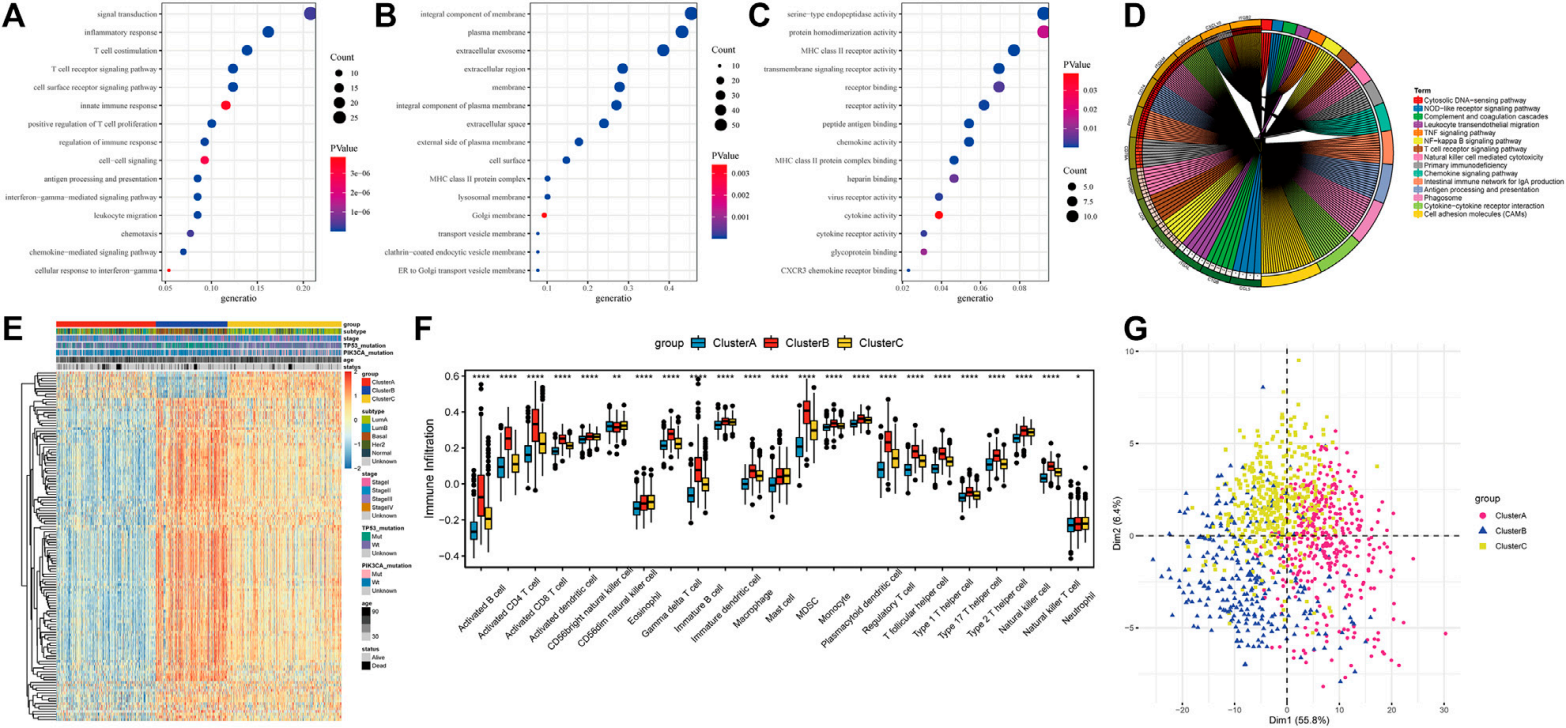
Differentially expressed genes (DEGs) identification and functional analysis among tumor subtypes. **(A)** Biological process(BP) for DEGs; **(B)** Cellular component(CC) for DEGs; **(C)** Molecular functions(MF) for DEGs; **(D)** The top 15 enriched KEGG pathway of DEGs; **(E)** Heatmap of differentially expressed gene; **(F)** Difference of immune cell infiltration in three subtypes; **(G)** PCA analysis of expression profile.

Based on the expression levels of 20 PR regulators, three subtypes of PR patterns were identified using consensus clustering analysis (Figures 3A–E). As shown in Figures 3F–J, the prognostic advantages of clusters B and C were better than those of cluster A. To explore the differences in biological behaviors among these three patterns, DE analysis was performed using the “limma” package of R software (Ritchie et al., 2015). We identified 979 and 235 DEGs in Cluster A compared to Cluster B and Cluster C, respectively, with *p* < 0.05 and | log2 (fold change) | > 1. Moreover, 132 DEGs were confirmed at the intersection of these two sets. To further identify the biological characteristics of these 132 DEGs, GSVA was performed, including KEGG and Gene Ontology (GO) enrichment analyses, and the bubble diagram of the top 15 enriched pathways was obtained in three classifications (BP, CC, and MF) (Figures 4A–C). The results showed that the DEGs were mainly enriched in the cell surface, plasma membrane and lysosomal membrane and were involved in biological processes, such as immune response, inflammatory response, T-cell receptor, antigen processing and presentation, and cell proliferation through receptor binding, chemokine activity and peptide antigen binding. In addition, KEGG enrichment analysis indicated that the intersected DEGs were mainly enriched in immune-related and tumor-promoting pathways, such as antigen processing and presentation, cell adhesion factors, phagosomes, primary immunodeficiency, chemokines, tumor necrosis factor, NF-kappa B signaling pathway, T-cell receptors, and other signaling pathways (Figure 4D). Heat map of differentially expressed genes among 3 Clusters (Figure 4E). Furthermore, ssGSEA results revealed that immune cell infiltration was significantly lower in Cluster A, such as activated B cells, activated CD8 T cells, activated CD4 T cells, immature B cells and macrophages (Figure 4F). Principal Component Analysis (PCA) among 3 Clusters (Figure 4G). All results suggested that the poor prognosis of Cluster A resulted from reduced immune cell infiltration.

### Construction of the Prognostic Model

To further reveal the role of PR-related lncRNAs in the prognosis and treatment of BC, Pearson’s correlation analysis (*p* < 0.001, |R| > 0.4) was used to assess PR regulators and their associated lncRNAs in BC, and 340 lncRNAs were screened out. In addition, we screened 867 DE lncRNAs associated with BC lung metastasis (*p* < 0.05) in nine paired BC samples with lung metastasis from the TCGA-BRCA cohort. Lastly, a total of 97 lncRNAs were used for further analysis in these DE lncRNAs.

**FIGURE 5:**
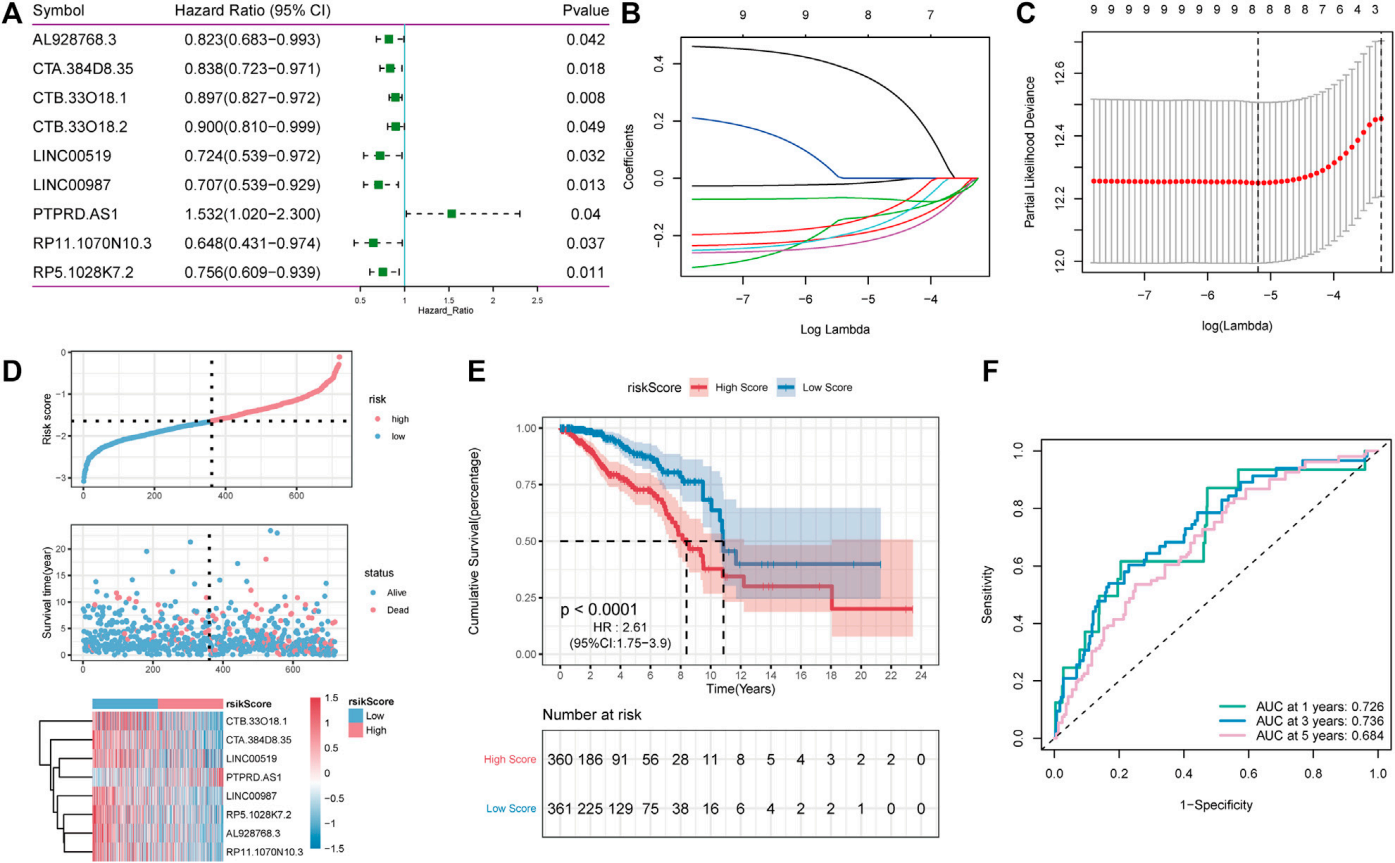
LncRNA Screening and construction of risk model. **(A)**: One-way cox screening for 9 lncRNAs; **(B)**: The change trajectory of each independent variable, with the horizontal axis representing the log value of the independent variable lambda and the vertical axis representing the coefficient of the independent variable; **(C)** Confidence interval under each lambda; **(D)**: Distribution diagram of risk score; **(E)**: OS curves for the different RP-score subgroups with the cut-off value; **(F)**: The time-dependent receiver operating characteristic (ROC) analysis of the RP-score. The area under the curve (AUC) was 0.726, 0.736, 0.684 at 1, 3, and 5 years, respectively.

Patients in TCGA-BRCA (*n* = 1,082) cohort were randomly divided into inner-training (*n* = 721) and inner-testing groups (*n* = 361) in a 2:1 ratio. Subsequently, in the training set, univariate Cox analysis was used to identify 97 candidate lncRNAs. The significance threshold was set to *p*-value < 0.05, and nine lncRNAs were retained (Figure 5A). Thereafter, the LASSO Cox regression model was used to construct a prognostic model based on the expression data of nine lncRNA genes in the TCGA training set. The model identified eight lncRNAs based on the optimal λ value of −5.2157 (Figures 5B,C). The formula of 8-lncRNA gene signature is as follows:

RS = (−0.018) * AL928768.3 + (−0.196) * CTA. 384D8.35 + (−0.138) * CTB. 33O18.1 + (−0.187) * LINC00519 + (−0.226) * LINC00987 + (0.371) * PTPRD.AS1 + (−0.148) * RP11.1070N10.3 + (−0.068) * RP5.1028K7.2.

To estimate the effects of the RS model on OS, patients with BC were divided into high- and low-RS groups based on the median value. As shown in Figures 5D,E, survival analysis using the Kaplan–Meier curves suggested that the OS of the high-RS group was significantly lower than that of the low-RS group (log-rank test, *p* < 0.001). Altogether, these results suggest that the RS model achieved satisfactory accuracy in predicting OS in the TCGA-BRCA dataset, and the area under the curve (AUC) at 1, 3, and 5 years was 0.726, 0.736, and 0.684, respectively (Figure 5F).

**FIGURE 6:**
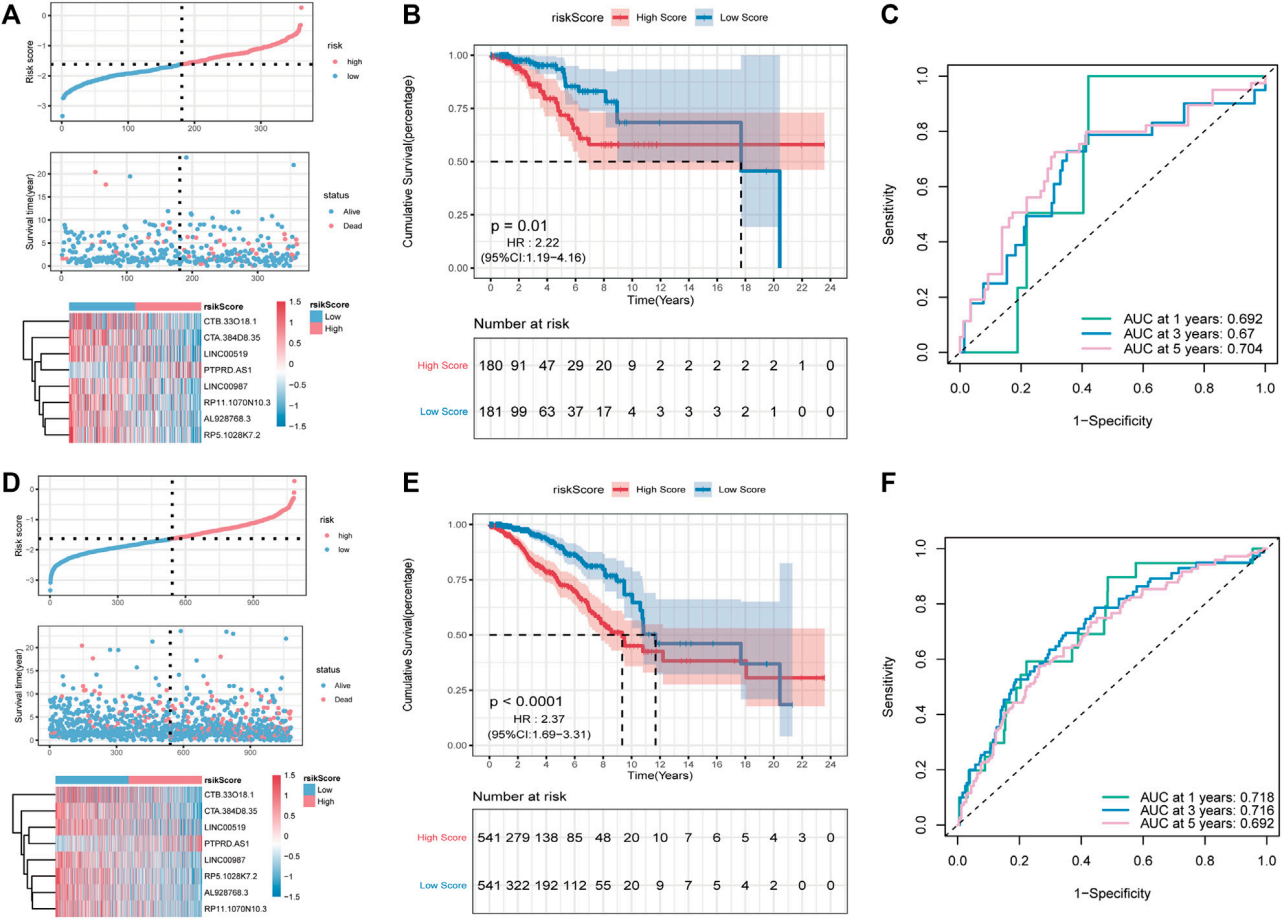
Validation of the PR-Score model in the overall set and validation set. **(A–C)** The distributions of risk score, survival status, ROC curve in the validation set. **(D–F)** The distributions of risk score, survival status, ROC curve in overall set.

Subsequently, the predictive ability of the RS model was validated for OS in both inner-training and testing groups. First, RS was calculated in both groups using the same algorithm. Thereafter, samples were divided into high- and low-risk groups according to the RS median, and a higher proportion of high-risk death samples could be observed (Figures 6A,D). Between-group analysis using the Kaplan–Meier curve showed that the OS in the high-RS group was significantly lower than that in the low-RS group (Figures 6B,E). As shown in Figure 6C, the RS model can accurately predict the OS, and the AUC values at 1, 3 and, 5 years were 0.692, 0.670, and 0.704, Figure 6C respectively, in the validation group and 0.718, 0.716, and 0.692, Figure 6F respectively, in the overall group.

**FIGURE 7:**
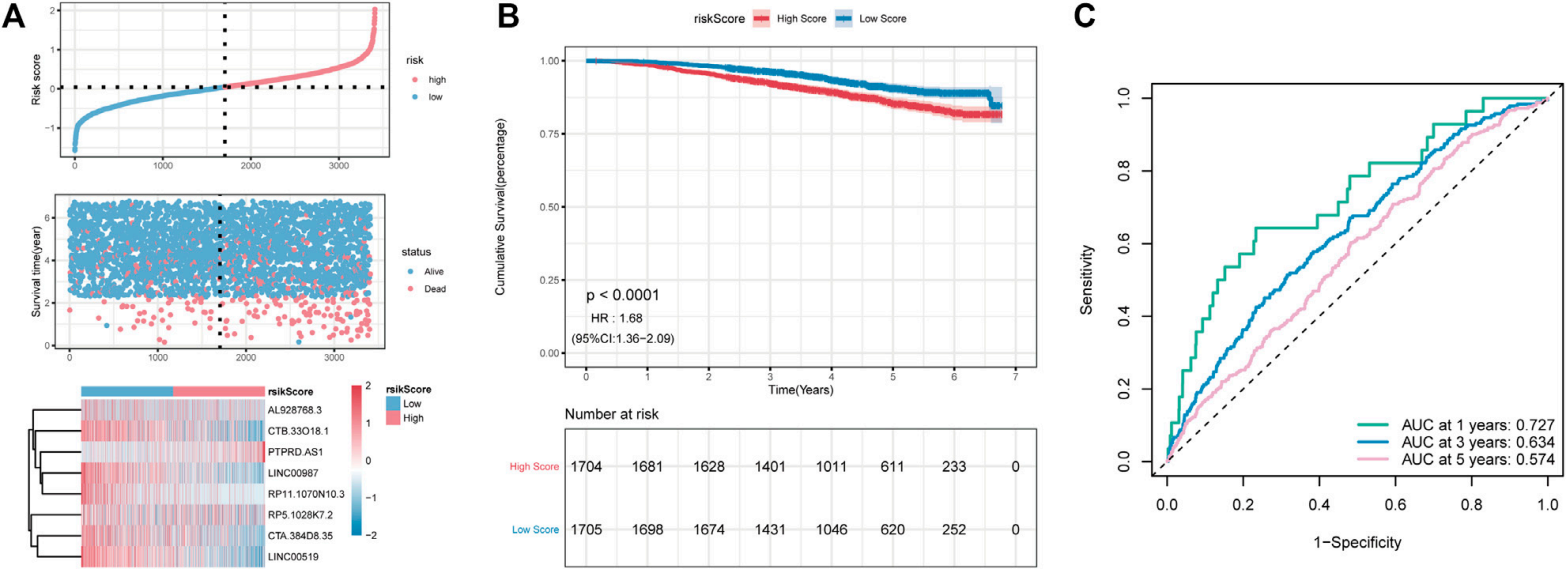
The external dataset GSE96058 validates the risk model. **(A)**: distribution diagram of risk score; **(B)**: survival curve; **(C)**: ROC curve at 1, 3, and 5 years.

To further assess the robustness of RS constructed using eight lncRNAs to predict the OS, GSE96058 was selected as the independent validation cohort. Using the formula mentioned above, the RS of GSE96058 samples was calculated, and the samples were divided into high- and low-risk groups according to the median. As shown in Figure 7A, a higher proportion of high-risk death samples could also be observed in GSE96058. The Kaplan–Meier curve showed that the OS in the high-RS group was lower than that in the low-RS group (Figure 7B). In Figure 7C, the RS model presents good accuracy in predicting OS, with AUC values of 0.727, 0.634, and 0.574 at 1, 3, and 5 years, respectively, in the GSE96058 dataset.

### Low-RS Model Identified the Alleviation of Clinical Characteristics

**FIGURE 8:**
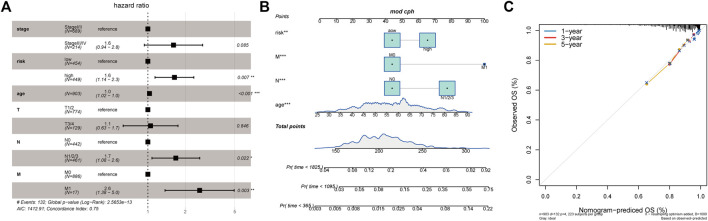
Relationship between tumor risk score and clinical characteristics. **(A)**: Multivariate Cox analysis of clinical characteristics and risk score; **(B)**: The nomogram of clinical characteristics and risk score; **(C)**: The validation plots of 1-, 3-, and 5-year nomogram.

Given the importance of the RS model in predicting the prognosis of patients with BC, we attempted to explore its value for clinical application. We assessed the RS as an independent prognostic factor distinct from age, stage, N-stage, T-stage, and M-stage using multivariate Cox analysis (Figure 8A). Nomogram is a powerful tool used to quantitatively estimate a personal risk by integrating multiple risk factors. Because N-stage, age, M-stage, and RS are predictors of OS in multivariate analysis, these variables were further included in the nomogram to predict the 1, 3, and 5-years OS of patients with BC. Figure 8B shows that the factors involved were assigned in proportion to its risk contribution to the survival. In addition, calibration curves showed good accuracy for predicting the OS at 1, 3, and 5 years in the BC cohort (Figure 8C). Altogether, these results suggest that a nomogram constructed using multiple factors has better capability to predict OS than a nomogram constructed using a single prognostic factor.

### RS Could Predict and Represent TMB

**FIGURE 9:**
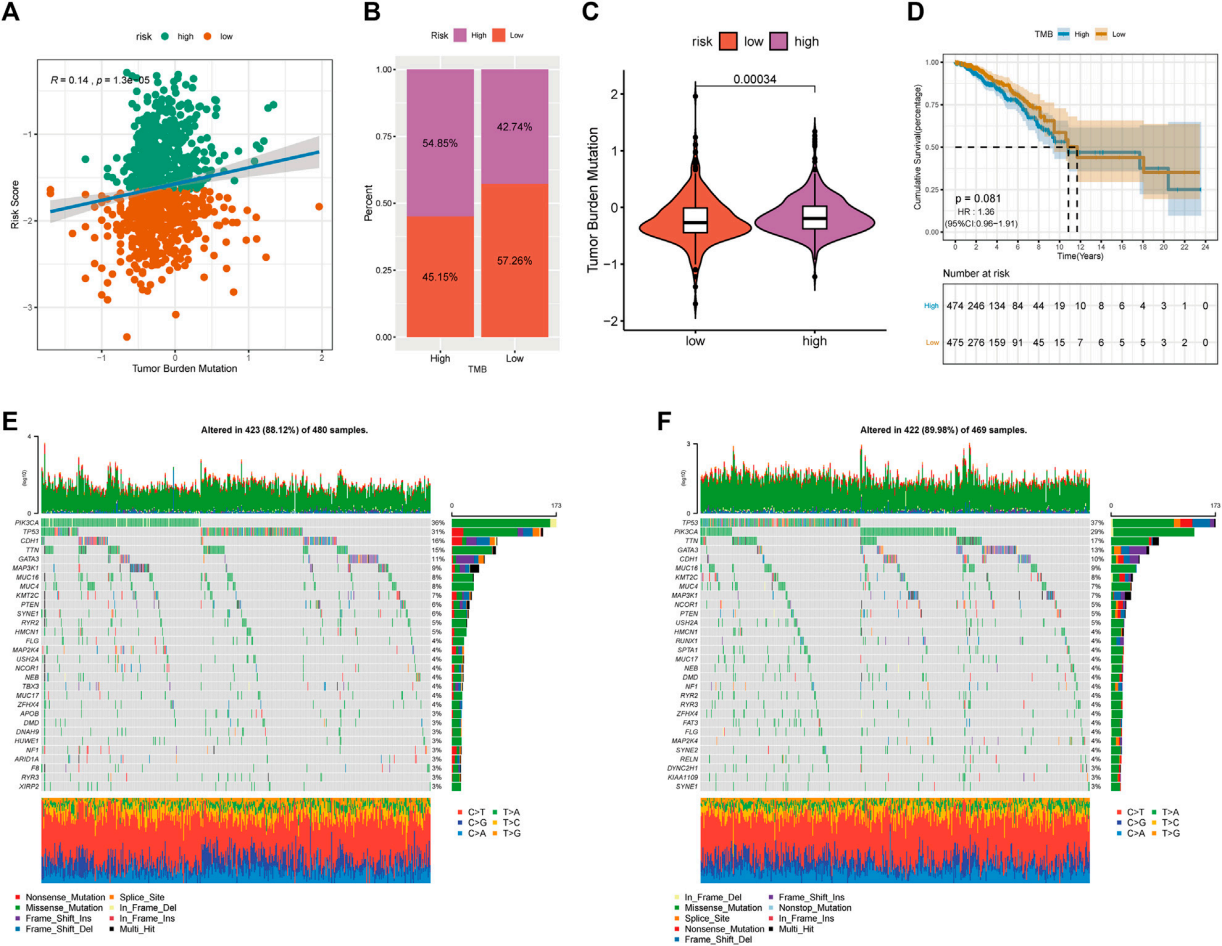
Relationship between tumor risk score and tumor mutation burden. **(A)**: correlation linear regression analysis; **(B)**: bar graph of proportion distribution; **(C)**: violin plot; **(D)**: survival curve; **(E)**: waterfall plot of gene mutation in low risk group; **(F)**: waterfall plot of gene mutation in high risk group.

Increasing evidence suggests that TMB may determine an individual’s response to immunotherapy. We further analyzed the characteristics of TME and RS of patients with BRCA. Two groups of high and low TMB were divided according to the median using the “maftools” package in R (Mayakonda et al., 2018), and differences in the survival time between the two groups were analyzed using KM. Furthermore, we found that TME was positively correlated with RS (Figure 9A). Figures 9B,C show that TMB was significantly higher in patients with high RS. However, no significant differences in survival time were observed in both groups (Figure 9D).

We further compared the top 30 driver genes with high frequency to evaluate the distribution of somatic variants in BRCA driver genes between low- and high-RS groups (Figures 9E,F). Differences in the mutation spectrum were found in both groups after being annotated from TCGA-BRCA files. Tumour PR-related patterns and TME status suggested the potential ability of immune checkpoints.

### Relationship Between Tumor RS and Immune Cell Infiltration

**FIGURE 10:**
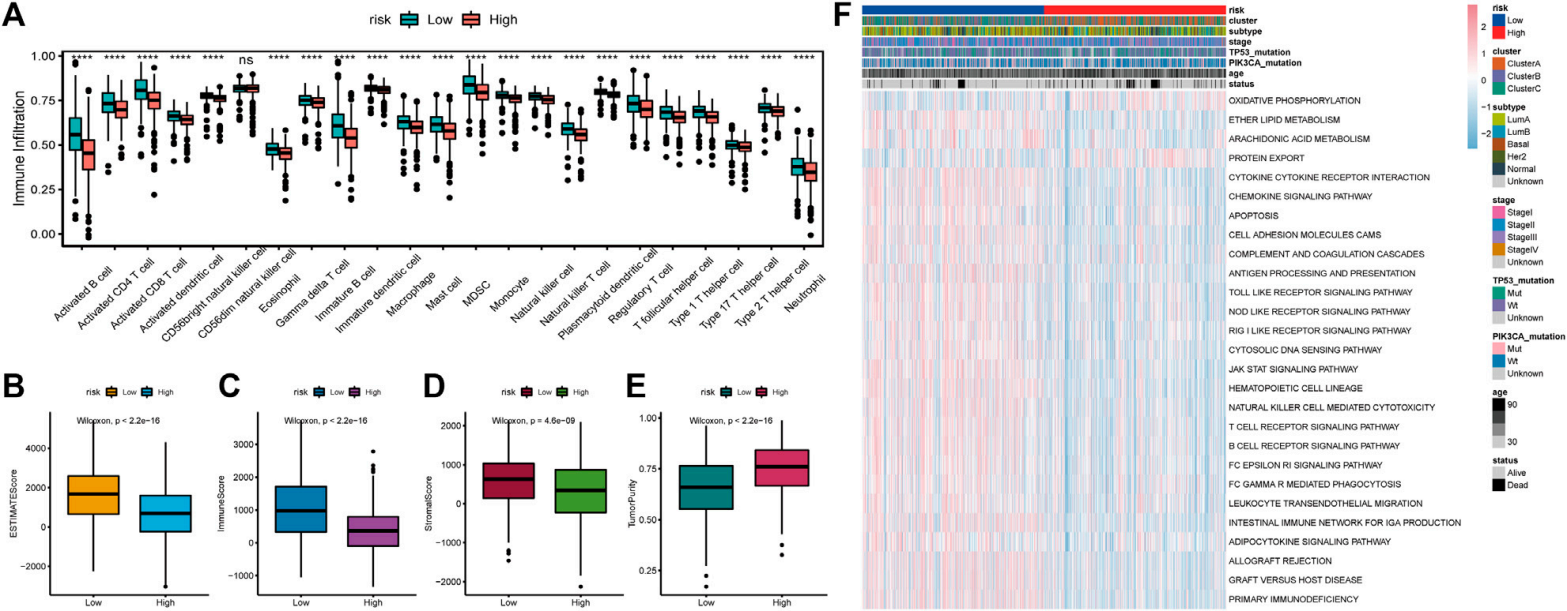
Relationship between tumor risk score and immune cell infiltration. **(A)**: Heat map of the distribution of immune cell infiltration ratio; **(B)**: difference of ESTIMATE Score; **(C)**: difference of Immune Score; **(D)**: difference of Stromal Score; **(E)**: difference of tumor purity; **(F)**: GSVA analysis of differentially expressed pathways.

We estimated the immune microenvironment in high- and low-RS groups. ssGSEA was used to assess the status of 23 immune cell infiltrates in the TCGA-BRCA cohort. As shown in Figure 10A, the infiltration abundance of most immune cells in the high-risk group was significantly lower, including activated B cells, activated CD8^+^ T cells, activated CD4 T cells (activated CD4 T cells), and immature B cells. The TME cell infiltration characteristics were calculated using ESTIMATE, including the tumor purity, stromal score and immune scores. The immune, stromal, and ESTIMATE scores were found to be significantly lower in the high-risk group, whereas the tumor purity was significantly higher (Figures 10B–E). The results of genomic variation analysis (GSVA) showed that metabolic pathways such as oxidative phosphorylation were more active in the high-risk group, whereas metabolic pathways such as antigen processing and presentation, apoptosis, B-cell receptor, T-cell receptor, and JAK-STAT were inhibited in the high-risk group (Figure 10F). These results suggest that decreased immune cell infiltration in the tumor microenvironment (TME) in the high-risk group indicates a worse immunotherapeutic response that may be responsible for the poor prognosis of patients.

### The Role of RS in Anti-PD1 Immunotherapy

**FIGURE 11:**
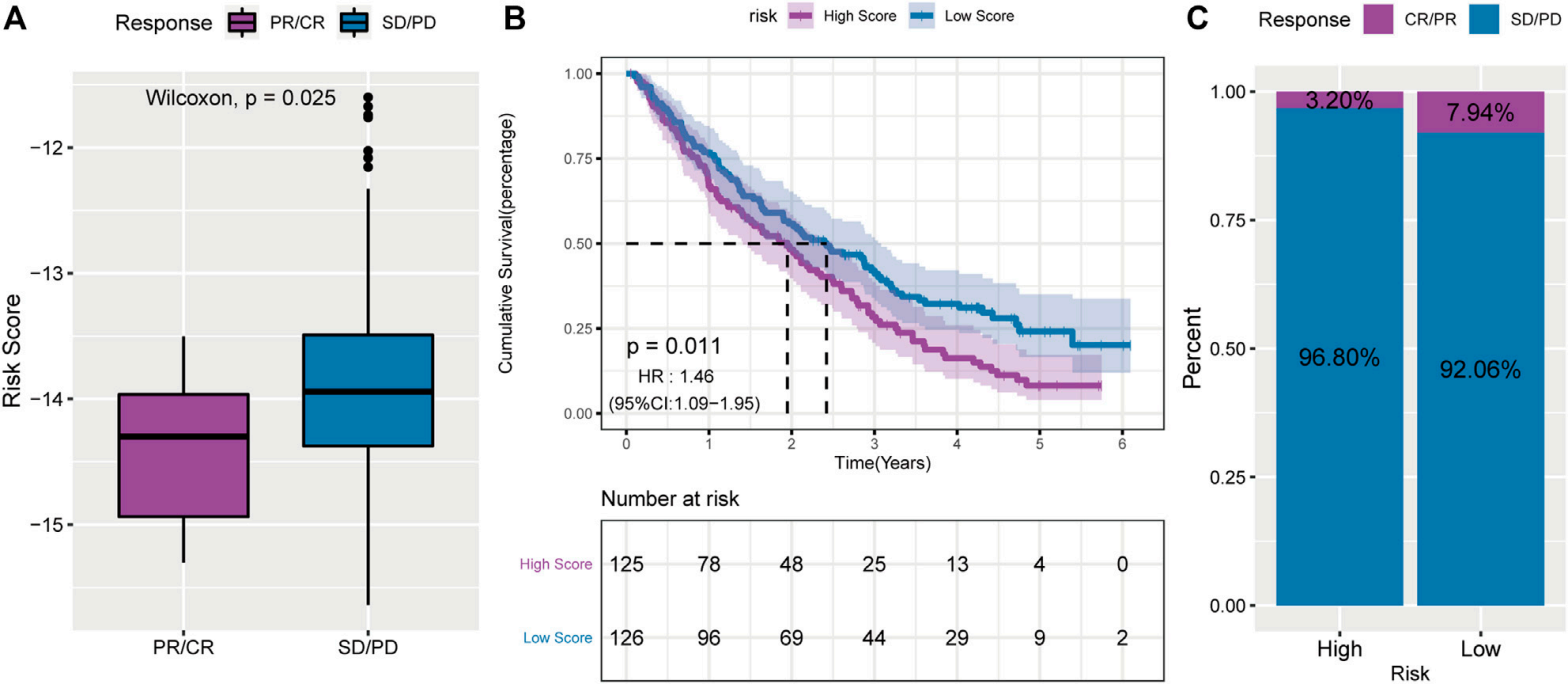
A powerful role of the PS-score scoring model in PD-1 blocking immunotherapy. **(A)**: comparison of risk scores of non-responsive group in PD-1 blocking therapy cohort; **(B)**: survival curve of high and low risk score groups in PD-1 blocking therapy cohort; **(C)**: proportion of patients with high and low risk score who respond to PD-1 blocking therapy.

Previous studies have shown a relationship between PD-L1 expression and PR (Zhang et al., 2020a). Therefore, we hypothesized that there might be a correlation between RS and immunotherapy. In the PD-1 blocking cohort, RS was lower in the responding group (CR/PR) than in the non-responding group (SD/PD) (Figure 11A). In addition, patients in the low-RS group lived significantly longer (Figure 11B) and had a higher objective response rate to PD-1 blockade therapy (Figure 11C). All these data demonstrated that the RS constructed by PR-related lncRNAs might correlate with immunotherapeutic responses.

## Discussion

PR is a novel type of programmed cell death occurring in cells infected by pathogens, as an embodiment of programmed cell death, thus stimulating the body’s inflammatory response. IL-18, IL-1β, and other inflammasomes are crucial elements in PR. Inflammasomes have been reportedly activated by NLRP3-ASC-caspase-1, promoting the expression of the downstream factors IL-18 and IL-1β and playing an anti-tumor role in colon cancer (Song and Li, 2018). Several studies have found that inflammatory response can promote tumor cell metastasis, and the production of inflammatory factors can also accelerate EMT, thus advancing tumor cell invasion and metastasis (Zaki et al., 2011). Simultaneously, simvastatin has been proven to help suppress the proliferation and metastasis of lung cancer *via* PR (Wang et al., 2018). However, its functions in the BRCA microenvironment and immune response remain elusive. Therefore, changes in the status and mechanisms of BC cells associated with the immune environment should be explored to guide clinical treatment targeting PR.

Therefore, we used eight PR-related lncRNAs to establish a prognostic model, which may provide a potential prediction for BC therapy targeting PR. First, three subtypes of pyroptotic tumors were identified based on the expression of 20 PR genes. These three subtypes had a significantly distinct prognosis, immune cell infiltration and molecular characteristics. These subtypes were also significantly related to immune activation, confirming the significant role of PR in immune regulation in TME landscapes. Subsequently, we classified PR Cluster A as having reduced immune cell infiltration and immune activation phenotype, with a lower survival disadvantage. We also investigated all pathways directly related to PR and explored a prognostic signature by analyzing the influence of the involved pathways on TME.

To provide a novel theoretical basis for the clinical practice of BC, a reliable risk assessment tool was established based on three PR subtypes. RS considered the heterogeneity of patients and associated PR with clinical prognosis. The low-RS group exhibited a more abundant immune cell infiltration and a longer OS rate. Previous studies have reported tremendous infiltration of inflammatory cells in the TME of BCRA, with the infiltration of CD8^+^ and CD4^+^ T cells remarkably associated with the prognosis of BRCA (Qian et al., 2011; Reddy et al., 2019). Moreover, cancer and immune-related pathways were found to be significantly enriched in the low-RS group. Furthermore, immune cell infiltration was significantly lower in Cluster A, including activated B cells, activated CD8 T cells, activated CD4 T cells, immature B cells, and macrophages. Similarly, compared with the high-RS group, the low-RS group was more sensitive. We believe that PR RS can be used as a prognostic prediction method to assess patient survival and efficacy of clinical response to immunotherapy.

Eventually, some limitations need to be addressed. Although multiple databases were used, namely, TCGA-BRCA and GEO, for verification, more information is required. All database searches were retrospective and lacked complete clinical information. Therefore, prospective studies and subgroup validation should be conducted. Furthermore, studies reporting on the role of PR in BC are limited, and our study can only provide preliminary theoretical support for future experimental verification. The risk model developed in this study did not exhibit a better predictive value for the OS of patients with BC, and the random survival forest algorithm exhibited overfitting and high variance. We plan to implement a more suitable machine learning method to improve the predictive ability.

## Conclusion

The risk model constructed based on eight lncRNAs co-expressed by PRGs, which were associated with lung metastasis of BC, was found to better determine the OS of BRCA tumor samples, which is an independent prognostic indicator of the clinical features of BRCA and to assess the benefits of immunotherapy.

## Data Availability

The datasets presented in this study can be found in online repositories. The names of the repository/repositories and accession number(s) can be found in the article/Supplementary Material.

## Supplementary Material

Supplementary Figure S1 Waterfall plot of gene mutations in TCGA-BRCA dataset.

Supplementary Figure S2 Effectsof TP53 gene mutation on apoptosis-related genes.

Supplementary Figure S3 Effectsof PIK3CA gene mutation on apoptosis-related genes.
