# Supplementary figures and images for: Pyroptosis-Related lncRNAs for Predicting the Prognosis and Identifying Immune Microenvironment Infiltration in Breast Cancer Lung Metastasis
Source: Front Cell Dev Biol. 2022 Mar 4;10:821727. doi: 10.3389/fcell.2022.821727 (PMC8931340; doi:10.3389/fcell.2022.821727)

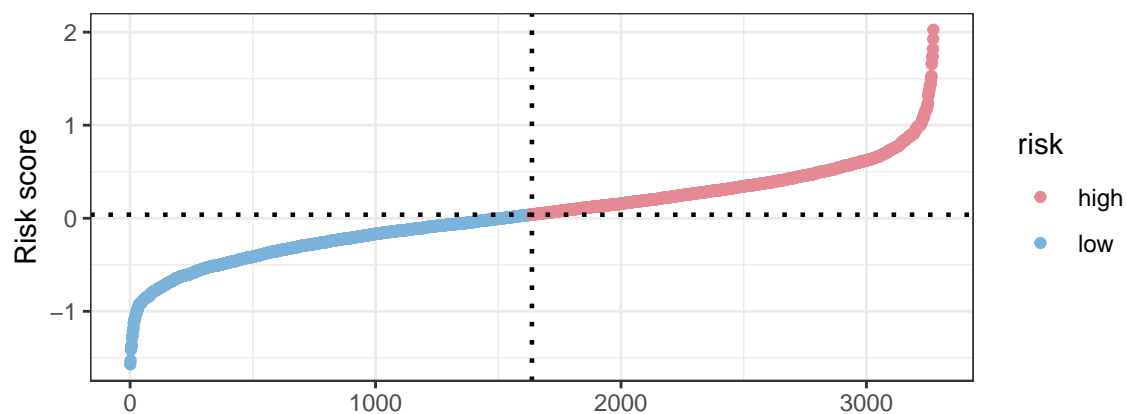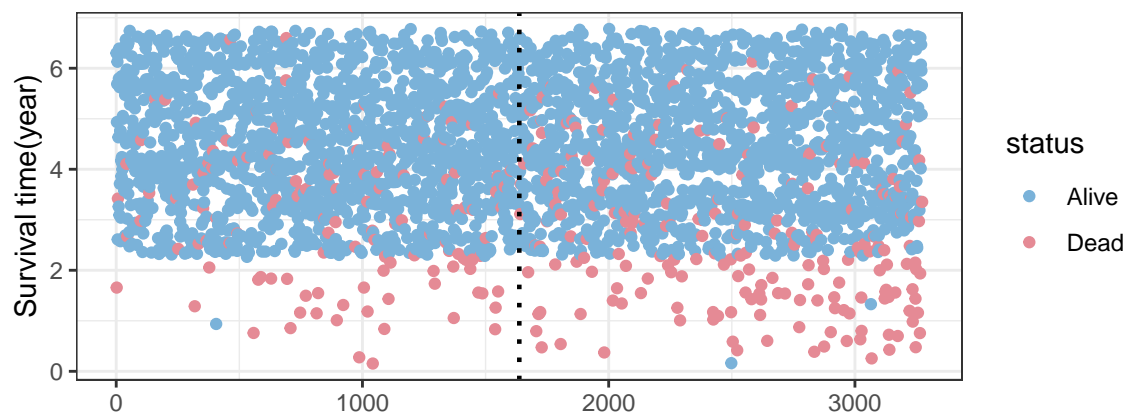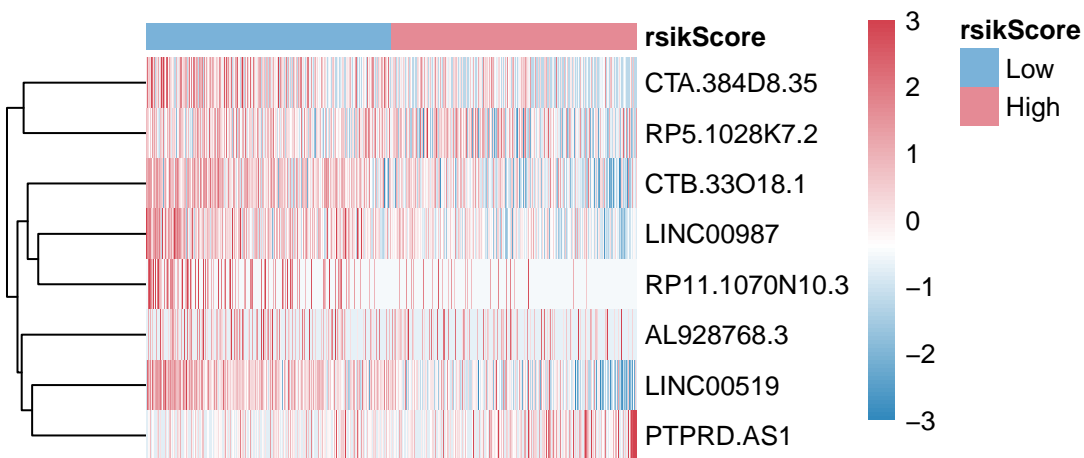

Supplement: Supplementary file 1 [file DataSheet2.PDF]

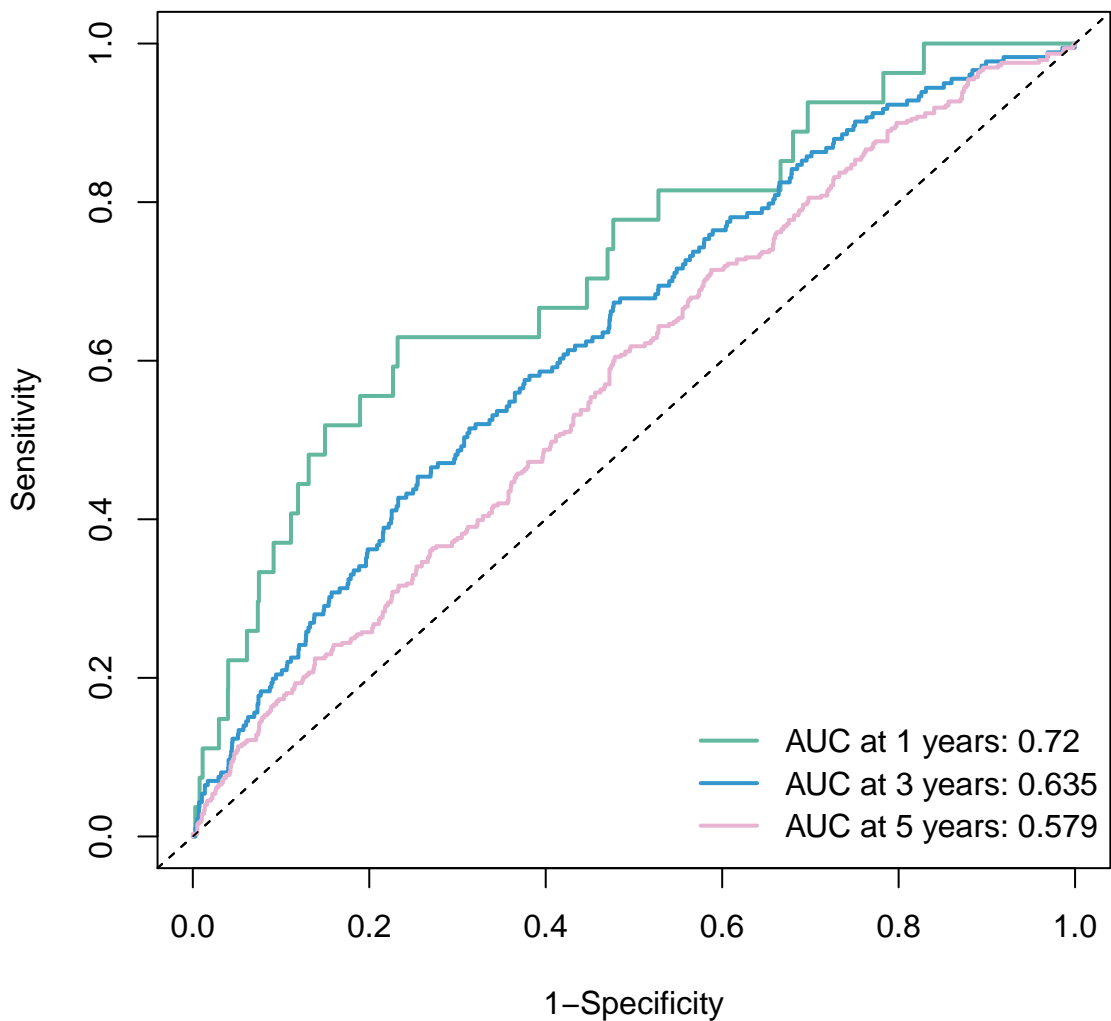

Supplement: Supplementary file 6 [file DataSheet1.PDF]
