# Supplementary material for: Pyroptosis-Related lncRNAs for Predicting the Prognosis and Identifying Immune Microenvironment Infiltration in Breast Cancer Lung Metastasis
Source: Front Cell Dev Biol. 2022 Mar 4;10:821727. doi: 10.3389/fcell.2022.821727 (PMC8931340; doi:10.3389/fcell.2022.821727)
Supplement: Supplementary file 5 [file DataSheet3.PDF]

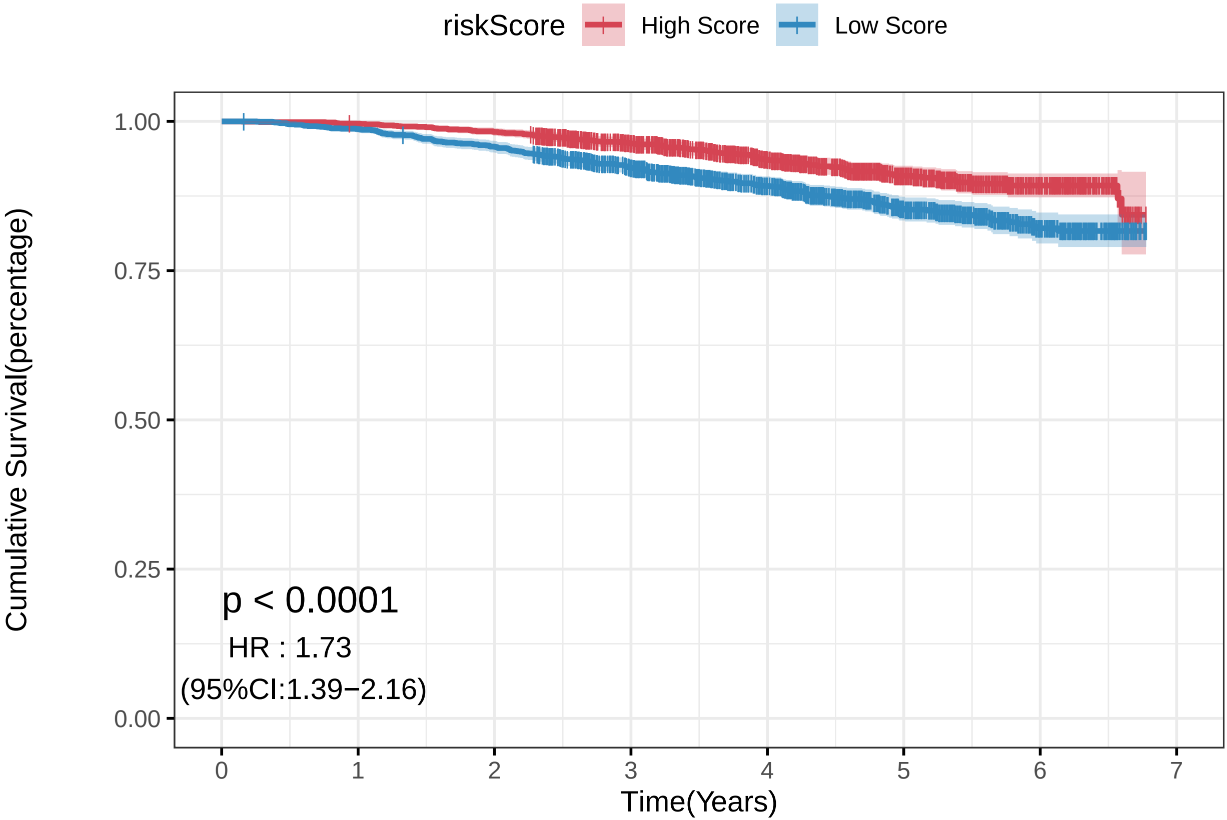

Number at risk

	0	1	2	3	4	5	6	7
High Score	1637	1630	1607	1371	995	590	242	0
Low Score	1636	1614	1565	1348	963	582	223	0
